# It is time to implement One Health approach to address health complex challenges!

**DOI:** 10.11694/pamj.supp.2015.22.1.6243

**Published:** 2015-10-10

**Authors:** Serge Nzietchueng

**Affiliations:** 1 Epidemiology and Veterinary Public Health Association (ESPV), Yaoundé, Cameroon

**Keywords:** EVD, capacity building, One Health, workforce

## Opinion

Africa's population is growing at the fastest rate and it is estimated that by 2100, 38.5% of the 10.9 billion humans will be leaving in Africa. [1]. Taking into consideration those projections and the current status of the health system in most countries of Africa, it is clear that, if nothing is done, some of these countries will face complexes health challenges. These challenges will be related to urbanization, pollution, climate change, land overuse, food security and safety, emerging and re-emerging communicable diseases, increase burden of non-communicable diseases and antibiotic resistance. Health systems and health services delivery will be put under extreme pressure, with potential negative impacts on Africa's human capital and overall development. The current Ebola outbreak in West Africa provides a good example of how disease outbreak can severely disrupt all aspects of the socio-economic fabric of a country with impact felt in areas as varied as social order, economic development, and food security [2, 3]. This represents an emerging and complex health challenges calling for changes in the current models of training and implementing public health policies in African countries. Training institutions in Africa should rethink their approach to training of the future public health workforce with focus on the acquisition of comptencies fit for current and future complex emergencies. A solid understanding of cross discipline and sector collaboration are key in addressing complex challenges; a move towards a good understanding of the broader concept of optimal health is now required, as stated in the definition of “health” by the World Health Organization (WHO) as a “a state of complete physical, mental and social well-being, not merely the absence of disease or infirmity” [4].

**Figure 1:**
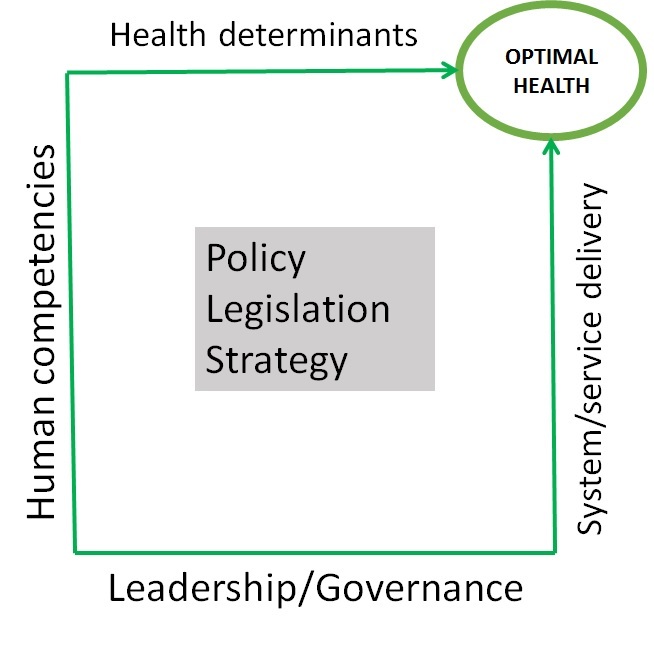
Interrelated elements which contribute to reach optimal health

The optimal health of a community is a result of a dynamic and fragile equilibrium among several elements such as: health determinants, human competencies, leadership, governance, health system, health delivery, policy and legislation (Figure 1). The current health workforce and health decision makers are not trained to perceive and understand the interaction and dynamics among health determinants, human competencies, leadership-governance, system-service delivery and policy to achieve optimal health. Furthermore, there is no emphasis on the mutual benefit of co-learning from each discipline, role, responsibility and contribution of the other disciplines or sectors to achieve the optimal health. That gap in the training of the current health work force and health decision makers represent a threat for achieving individual and community optimal health. Besides that, the current Ebola outbreak gives us a strong evidence that a fire-fighter strategy which has mobilized sympathy of the international health community and pull a huge among of money is not a pro-active strategy and may not be the best approach to address complex health challenges. As health actors, we have an opportunity to rethink our training curricula and model, leadership, policy, governance, health system and service delivery. We should look toward a long term and dynamic One Health training program, strategy, policy and legislation in order to anticipate future health challenges.
